# Bactericidal human monoclonal antibody 1B1 shows specificity for meningococcal factor H binding protein variant 2 and displaces human factor H

**DOI:** 10.1096/fba.2023-00077

**Published:** 2024-06-27

**Authors:** Daniele Veggi, Chelsy C. Chesterman, Laura Santini, Ying Huang, Nicola Pacchiani, Jeannette Sierra, Lynn Chen, Jason Laliberte, Federica Bianchi, Roberta Cozzi, Elisabetta Frigimelica, Domenico Maione, Oretta Finco, Matthew J. Bottomley

**Affiliations:** ^1^ GSK Siena Italy; ^2^ GSK Rockville Maryland USA

**Keywords:** antibody specificity, factor‐H binding protein, meningococcus, serum bactericidal assay, vaccine, x‐ray structure

## Abstract

Thousands of disease cases and hundreds of deaths occur globally each year due to invasive meningococcal disease. *Neisseria meningitidis* serogroup B (MenB) is the leading cause of such disease in developed countries. Two vaccines, 4CMenB and MenB‐fHbp, that protect against MenB are available and include one or two forms respectively of factor H binding protein (fHbp), a key protective antigen. Studies of circulating meningococci have identified over 1380 different fHbp amino acid sequences, which form three immunologically distinct clusters, termed variants 1, 2, and 3. Neither of the current vaccines contains a variant 2 antigen, which is less well characterized than fHbp variants 1 and 3. We characterized the interaction of fHbp variant 2 with humAb 1B1 using biochemical methods and live meningococcal assays. Further, we determined the crystal structure of the complex at 2.4 Å resolution, clearly revealing the epitope and providing the first detailed report of an antibody with distinct specificity for fHbp variant 2. Extensive mutagenesis and binding studies elucidated key hotspots in the interface. This combination of structural and functional studies provides a molecular explanation for the bactericidal potency and specificity of humAb 1B1 for fHbp variant 2. Our studies, focused on fHbp variant 2, expand the understanding of this previously under characterized group of the vast family of variants of fHbp, a virulence factor present on all meningococci. Moreover, the definition of a protective conformational epitope on fHbp variant 2 may support the design and development of novel variant 2‐containing MenB vaccines affording greater breadth of protection.

## Graphical abstract

We performed a structural and functional characterization of human monoclonal antibody 1B1. x‐ray crystallographic studies revealed the molecular basis of the distinct specificity of 1B1 for factor H binding protein (fHbp) variant 2. Understanding the epitope/paratope interface shows how 1B1 displaces human factor H from fHbp variant 2, which contributes to the high potency of antibody 1B1 in the killing of serogroup B meningococcus (MenB).

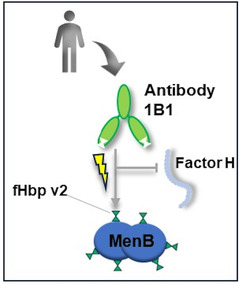

## 1 INTRODUCTION

Thousands of disease cases occur annually due to *Neisseria meningitidis* infections.^1^, ^2^ Invasive meningococcal disease is highly feared because it can rapidly be fatal or cause severe long‐term sequelae such as limb amputation, hearing, and visual impairment, cognitive dysfunction, seizure, and behavioral disorders.^3^, ^4^ For over a decade, licensed glycoconjugate vaccines have effectively protected against meningococcal serogroups A, C, W135, and Y.^5^, ^6^ More recently, protection against meningococcal serogroup B (MenB) became possible through the development and licensure of two different recombinant protein vaccines (4CMenB and MenB‐fHbp).^7^, ^8^, ^9^ Real‐world data reviewed after millions of 4CMenB vaccinations in eligible infants in England showed high safety and effectiveness in preventing MenB disease.^10^, ^11^, ^12^

The two licensed MenB vaccines both contain factor H binding protein (fHbp), a key immunogen eliciting antibodies that exert complement‐mediated bactericidal activity.^7^, ^13^ Meningococcal fHbp is a 28 kDa surface‐exposed lipoprotein present on all known strains of *N. meningitidis*; its 3D structure comprises two β‐barrel domains connected by a short linker.^14^, ^15^, ^16^, ^17^ During meningococcal infections, fHbp binds the human complement regulatory factor H (hfH) thereby coating the bacterial surface, inhibiting activation of the complement alternative pathway and enabling evasion of host innate immunity.^18^, ^19^ Hence, the meningococcal virulence factor fHbp is an important target of vaccine‐elicited protective antibodies.

Over 1380 different amino acid sequence variants of fHbp have been reported to date,^20^ and they cluster phylogenetically as three variants.^21^ The three variants are immunologically distinct, that is one fHbp variant typically induces antibodies with high bactericidal activity against strains expressing similar variants (usually sharing ≥80% fHbp sequence identity), but with minimal activity against strains expressing different fHbp variants with lower sequence identity. The notable sequence variability of fHbp led vaccine developers to include multiple antigens in the MenB formulations: 4CMenB contains four different components (hence 4C) including fHbp variant 1, while MenB‐fHbp contains two components: fHbp variants 1 and 3. Neither vaccine contains fHbp variant 2, suggesting the possibility that vaccine efficacy might be improved by inclusion of a variant 2 antigen. To potentiate the design and development of such a vaccine, we sought to deepen our understanding of fHbp variant 2 structure and antigenicity.

Since 2018, crystal structures have been reported for fHbp variant 1 in complex with cross‐reactive human monoclonal antibodies (human mAbs, or humAbs) 1A12^22^ and 4B3,^23^ and for fHbp variant 3 in complex with humAb 1E6,^24^ collectively revealing the specificity and mechanism of action of these humAbs. However, to date, there are no reported structures of any mAbs bound to fHbp variant 2, and our structural understanding of wild type fHbp variant 2 is limited to its C‐terminal β‐barrel domain only, determined previously by x‐ray crystallography.^25^ The structure of an unnatural mutated form of fHbp variant 2 with increased thermostability was also determined previously.^26^ It is likely that the unusually low thermostability of the N‐terminal domain (NTD) of wild type fHbp variant 2^25^, ^27^ hampered prior efforts to determine its structure. Consequently, in addition to lacking a wild type variant 2 structure encompassing its NTD, there are also currently no well‐defined epitopes specific to variant 2. Such knowledge gaps should be filled since both antigen structure and antigen‐specific mAbs can provide crucial information and act as key reagents during vaccine development.^28^, ^29^, ^30^

We previously reported a study of samples collected from three healthy adult 4CMenB vaccinees, in which we identified and characterized a total of 110 different humAbs (where each humAb was obtained from a single individual). Therein, we principally focused on a remarkable cross‐reactive humAb 1E6.^24^ However, that study also identified humAb 1B1, and in a qualitative binding assay using recombinant Fab produced in *E. coli*, 1B1 appeared to show specificity for fHbp variant 2 (see Figure 1 in Bianchi et al.^24^). To date, there are no other reports of a humAb with specificity for fHbp variant 2, and only one other antibody (JAR11, from mouse) with variant 2 specificity has been reported, for which little epitope mapping information was provided.^31^, ^32^ While it was unexpected to obtain from 4CMenB vaccinees a mAb with specificity for fHbp variant 2, it is readily conceivable that the donor from whom we identified mAb 1B1 might have been previously infected with a meningococcal strain expressing fHbp variant 2, thus giving rise to the corresponding B cell. Here, we report the first detailed characterization of the bactericidal humAb 1B1 and its structure in complex with wild type fHbp variant 2. We used a variety of biochemical and live meningococcal assays. Coupled with x‐ray crystallographic and structure‐guided mutagenesis studies, we provide insights into the structure of wild type fHbp variant 2, its recognition by humAb 1B1, and the molecular basis underlying the distinct variant 2 specificity observed.

## 2 MATERIALS AND METHODS

### 2.1 Molecular cloning, protein expression, and purification of wild type proteins

All wild type fHbp proteins were cloned, expressed and purified as described previously. The human antibody 1B1 was cloned, expressed and purified in both Fab and full‐length IgG1 formats, as described previously.^24^

### 2.2 Human samples

As described previously,^24^ the human samples from which 1B1 and 4B3 antibody sequences were derived were collected from three healthy adults immunized with the 4CmenB vaccine, in a clinical trial conducted in Krakow (Poland), approved by the Bioethics Committee of the District Medical Doctors Chamber in Krakow and conducted in accordance with the Declaration of Helsinki. The use of samples was performed upon written informed consent obtained from participants before the study‐specific procedures.

### 2.3 Site‐directed mutagenesis of fHbp proteins

Oligonucleotide primer pairs designed in‐house and synthesized externally (IDT) were used for site‐directed mutagenesis PCR reactions (NEB) to introduce mutations of interest into the appropriate template plasmid encoding fHbp variant of interest (pET‐21b‐v1.1, pET‐21b‐v2.16, or pET‐21b‐v3.28). Single colonies were picked, cultured, and plasmid DNA extracted for sequence confirmation.

### 2.4 Production and characterization of fHbp mutants for BLI binding studies

Plasmids encoding fHbp mutants were transformed into BL21 (DE3) Star cells (Novagen) according to the manufacturer's protocol. Three colonies were transferred into 500 μL of TB media in 24‐deep well blocks. Cultures were grown for 2–4 h at 37°C with shaking at 250 rpm. Cultures were diluted with 3.5 mL of TB media and transferred to 25°C for 30 min. 1 mM IPTG was added before overnight incubation of the cultures. Harvested cell pellets were re‐suspended in 750 μL of BugBuster MasterMix (Sigma). Protein was purified through parallel one‐step nickel affinity His‐tag purification on a Kingfisher instrument (ThermoFisher) using MagHis particles (Promega). Protein was bound to 100 μL of suspended MagHis particles, washed three times in 25 mM Tris pH 8.0, 300 mM NaCl, 20 mM Imidazole, and eluted in 25 mM Tris pH 8.0, 300 mM NaCl, 250 mM Imidazole. Protein concentration was determined by biolayer interferometry (BLI) using Anti‐His (HIS2, Fortebio) biosensors operated at 30°C in an Octet 384‐Red instrument (Fortebio). Binding to purified fHbp was used to establish a standard curve for the quantitation experiments.

### 2.5 Biolayer interferometry binding studies of fHbp/mAb interactions

Binding affinity of fHbp variants 1.1, 2.16, 3.28, and mutants thereof, to mAb 1B1 or 4B3 was determined with an Octet instrument using Anti‐Fc Human (AHC, Fortebio) biosensors at 30°C. Wild type and mutant fHbp proteins were diluted in Octet buffer (1 × PBS, 1% (w/v) BSA) with protein concentrations ranging from 150 to 1 nM. Octet biosensors were first dipped in a solution of Octet buffer to establish the initial baseline and then mAbs were coated onto the biosensors by submersion in a 10 mcg/mL solution of antibody. Sensors were re‐equilibrated in Octet buffer before transfer of the tips to the fHbp serial dilution. The association binding curve was measured by the increase in Octet BLI signal over time, followed by a dissociation phase with the sensors transferred back to Octet buffer. Curves were manually inspected for quality and binding curves that contain only noise were removed, leaving a minimum of five curves for fitting. Binding curves for association and dissociation were fit with the ForteBio Analysis 11.0 software to a 1:1 binding ratio in order to determine the equilibrium dissociation constant (K_D_) of the interaction. K_D_ values reported are the average and standard deviation of three independent measurements.

### 2.6 Flow cytometry

The ability of 1B1 mAb to bind antigen exposed on the surface of *N. meningitidis* bacteria was determined using a FACScan flow cytometer (FACSCanto II, BD Biosciences, San Jose, CA, USA). Bacteria grown until early‐log phase (OD_600_ of ~0.25) were incubated with monoclonal antibody at the concentration of 10ug/mL. Antibody binding was detected using an Anti‐Human IgG (H + L)‐FITC conjugated produced in goat (Invitrogen 31,529) at a 1:100 dilution. Bacteria plus PBS‐1%BSA and secondary antibody were used as negative control. A total of 10,000 events for each sample was collected with the cytometer and FlowJo™ Software v.10 Flow Jo was used to analyze the data.

### 2.7 Inhibition of binding of fH

The ability of the HumAb to inhibit binding of fH to live bacteria was measured by flow cytometry. Bacterial cells grown until mid‐log phase (OD600 of ~0.5) were incubated with anti‐fHbp mAb (50 μg/mL in PBS‐1%BSA buffer) for 30 min at room temperature, followed by the addition of purified human fH (50 μg/mL for MC58 and M1239, 10 μg/mL for UK104), which was incubated for an additional 30 min at room temperature in a final reaction volume of 100 μL. fH binding was detected with a goat polyclonal antiserum to fH (Calbiochem 341,276) diluted 1:100 and incubated for 30 min at room temperature, followed by additional 30 min incubation with a donkey anti‐goat IgG–fluoresceinisothiocyanate (FITC) conjugate (Jackson Immunoresearch 705.095.003) diluted 1:100 in PBS‐1%BSA buffer.

### 2.8 Serum bactericidal assay

Bacteria grown until early log phase (OD_600_ of ~0.25) were diluted in Dulbecco's Phosphate Buffered Saline (DPBS) containing 1% bovine serum albumin (BSA) and 0.1% glucose at the working dilution of 10^4^–10^5^ and incubated with serial two fold dilutions of test monoclonal antibody starting from a concentration of 125 μg/mL. Serum bactericidal titers were defined as the monoclonal antibody dilution resulting in 50% decrease in CFU per milliliter after a 60‐min incubation of bacteria with the reaction mixture compared to the control CFU per milliliter at time zero. Pooled baby rabbit sera (Cedarlane, Burlington, Canada) or human serum obtained from volunteer donors under informed consent, were used as a complement source for rSBA or hSBA respectively.

### 2.9 Protein crystallization, diffraction data collection, and processing

To form the fHbp/Fab complex, 7 mg of fHbp variant 2.16 and 5 mg of Fab 1B1 were co‐incubated overnight at 4°C and further purified by size exclusion chromatography to remove excess protein using a prepacked HiLoad 26/60 Column Superdex 75 Preparation Grade (GE Healthcare). The fHbp/Fab complex at a concentration of 15 mg/mL in buffer containing 50 mM Tris–HCl pH 8.0 and 200 mM NaCl were screened in over 500 different crystallization conditions using prepacked 96 deep‐well blocks commercialized by Molecular Dimensions (Newmarket, UK) using a Crystal Gryphon robot (Art Robbins Instruments, Sunnyvale, CA, USA). After incubation at 21°C using a RockImager‐182 system (Formulatrix Inc.), crystals were obtained using a reservoir solution containing 0.09 M sodium Nitrate, Sodium Phosphate dibasic, Ammonium Sulphate, 0.1 M Buffer Imidazole, MES monohydrate, pH 6.5 and as precipitant mix 25% v/v MPD, 25% PEG 100, 25% w/v PEG 3350.

Crystals were soaked in the mother liquor supplemented with 15% ethylene glycol prior to cryo‐cooling in liquid nitrogen. Diffraction was tested at the ESRF, beamline ID30A‐1, and several full datasets were collected at 100 K, wavelength *λ* = 0.966 Å, on a Pilatus3_2M Detector (see Table 2). Diffraction datasets were indexed, integrated, and scaled with XDS^33^ and Aimless,^34^ via the CCP4 suite.^35^ Crystals of the fHbp‐1B1 complex belonged to space group P1211, with the asymmetric unit containing three complexes and having a solvent content of 52% (Matthews coefficient of 2.69 Å^3^/Da). The structure was solved by molecular replacement (MR) using Phaser software.^36^ The search model templates included three distinct structures. First, we used the fHbp variant 1 from a previously determined 2.0 Å‐resolution structure (pdb 3KVD).^15^ Prior to MR, the fHbp template was modified by mutation to alanine residues at several positions where fHbp variants 1 and 2 differ in sequence. Second, we included the variable region of the Fab 4B3 structure and, third, the constant region of the Fab 4B3 structure—where both chains were separately extracted from Fab 4B3 in the complex structure determined previously (pdb 6XZW).^23^ Again, prior to MR, the Fab‐derived search models were alanine‐mutated or trimmed in the more variable regions. Thus, three separate MR search models were used (rather than the entire structure of the previous fHbp/4B3 complex) in order to minimize risk of bias in the MR solution.

### 2.10 Structure refinement

Initial MR solutions were subjected to cycles of manual building in Coot^37^ and subsequently refinement with Phenix.refine.^38^ Structure figures were created with Pymol (www.pymol.org). Structure factors and atomic coordinates have been deposited in the Protein Data Bank (www.wwpdb.org)^39^ with accession code PDB 8BK2.

## 3 RESULTS

### 3.1 HumAb 1B1 shows high affinity and distinct specificity for fHbp variant 2

Therefore, here, we first sought to confirm the apparent specificity of humAb 1B1 in a quantitative binding analysis based on biolayer interferometry (BLI), using a full‐length IgG form of recombinant 1B1 produced in mammalian cells, which are generally considered a more appropriate expression system than *E. coli* for the faithful production of heterologous mammalian proteins (see Section 2).

**FIGURE 1:**
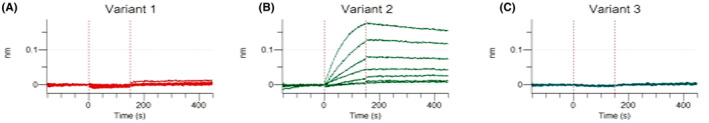
Biolayer interferometry (BLI) experimental data exploring the interaction of humAb 1B1 with fHbp variant 1 (panel A), fHbp variant 2 (panel B) and fHbp variant 3 (panel C). First, BLI biosensors suitable for capturing the Fc region of human antibodies were dipped into a solution of humAb 1B1 in order to coat the biosensor surface with antibody and reach equilibrium. Then, at the start of the binding experiment (Time = 0 (s)), the biosensors coated with humAb 1B1 were transferred into seven fHbp solutions representing a serial dilution of 150–1 nM fHbp antigen, for 150 s. Upon binding of any macromolecule to humAb 1B1, the BLI biosensors register an increase in the light interference pattern (y‐axis shows this increase as wavelength shift, in nm), proportional to the amount of antigen bound. After 150 s, the biosensors are transferred into antigen‐free buffer solution, resulting in dissociation of antigen from biosensors and a decrease in signal over time. Hence, the notable BLI responses observed only in panel B indicated binding to humAb 1B1 only for fHbp variant 2, and not for fHbp variants 1 or 3.

Antibody–antigen interactions were measured for humAb 1B1 captured via its Fc region, with fHbp proteins provided in solution. HumAb 1B1 showed strong interactions with fHbp variant 2 (equilibrium binding dissociation constant, K_D_, 0.24 ± 0.03 nM), while no interactions with fHbp variants 1 or 3 were detected (Figure 1). (We tested the often‐used fHbp exemplars (variants 1.1, 2.16 and 3.28), it cannot easily be excluded that there may exist some mutated forms of variants 1 or 3 that exhibit some affinity for humAb 1B1). These data confirm that humAb 1B1 has distinct specificity for fHbp variant 2 and forms a stable complex with moderately high affinity.

### 3.2 HumAb 1B1 preferentially binds and mediates bactericidal activity against live meningococci expressing fHbp variant 2

**FIGURE 2:**
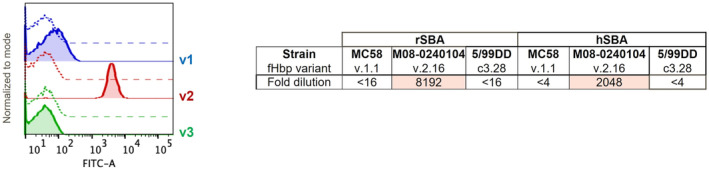
Flow cytometry data—the histograms display the frequency distribution of the bacteria (y‐axis) versus fluorescence intensity (x‐axis). The FITC‐fluorescence detected on the surface of bacteria after the incubation with 10 μg/mL of mAb 1B1 was acquired in logarithmic scale (blue line, MC58 strain; red line, M08‐0240104 strain; green line, M1239 strain). For each MenB strain, dotted line histograms represent negative control bacteria incubated with PBS and anti‐human IgG FITC‐conjugated. Only for fHbp variant 2 the peak fluorescence notably shifted to the right (fluorescence measurement >10^3^), indicating that mAb 1B1 binds significantly only to the meningococcal strain displaying fHbp variant 2.

Following the in vitro binding analysis, we examined the 1B1/fHbp interaction in a more physiological context, by monitoring binding of humAb 1B1 to the surface of live meningococci. Using flow cytometry, we found that humAb 1B1 bound extensively to MenB strain M08‐0240104 which expresses variant 2, but only at very low levels to strain MC58 (expresses variant 1) and not detectably to strain M1239 (expresses variant 3) (Figure 2).

We also examined whether the binding to bacteria matched the functional activity of humAb 1B1 in standard serum bactericidal activity (SBA) assays, the established surrogate of protection for *N. meningitidis*.^40^, ^41^ In SBA assays using either rabbit or human sources of complement, humAb 1B1 was highly effective at killing MenB strain M08‐0240104 (expresses variant 2) but was not effective at killing strains expressing variants 1 or 3 (Table 1). Overall, the observations from these in vivo experiments align with the in vitro binding study, demonstrating that humAb 1B1 shows distinct specificity for fHbp variant 2 in both biochemical and live meningococcal functional assays.

**TABLE 1:** SBA data showed 1B1 exerts bactericidal activity specifically against a MenB variant 2 strain.

	rSBA			hSBA		
MenB strain	MC58	M08‐0240104	5/99DD	MC58	M08‐0240104	5/99DD
fHbp variant	1.1	2.16	3.28	1.1	2.16	3.28
Titer	<16	8192	<16	<4	2048	<4

*Note*: Summary of serum bactericidal assay (SBA) data show that 1B1 is effective at killing MenB strain M08‐0240104 expressing variant 2, but not MenB strains expressing variants 1 or 3, either using rabbit (rSBA) or human (hSBA) complement. Values are expressed as titer, using 2‐fold serial dilutions from a mAb 1B1 stock at a concentration of 0.5mcg/mL. The strain 5/99DD is a complemented strain that expresses high amounts of variant 3.28 and is susceptible to killing in SBA assays, as reported previously.^42^ Hence, SBA data for strain 5/99DD, fHbp 3.28 complemented is shown, rather than for strain M1239 which naturally expresses fHbp v3.28 but which is known to be insensitive to killing in the SBA assay and indeed resulted negative (titers <16) when tested with humAb 1B1. The boxes reporting significatives hSBA titers (8192 and 2048) are emphasized by color shades.

### 3.3 HumAb 1B1 competes with human factor H for binding to meningococci expressing fHbp variant 2

**FIGURE 3:**
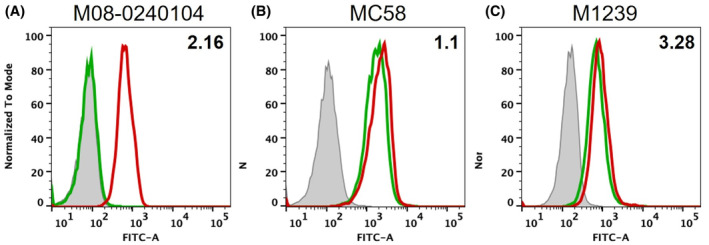
Flow cytometry data showing binding of human factor H (hfH) to live meningococci displayed as FITC‐fluorescence intensity in the x‐axis: the histograms represent the fluorescence of bacteria pre‐incubated with humAb 1B1 (green line) or with buffer only (red line). Gray‐filled areas represent negative control bacteria incubated without humAb 1B1 and hfH, only with buffer and secondary FITC‐conjugated antibodies. Panel A: for v2.16, hfH binding was detectable when the bacteria were pre‐incubated with buffer alone (red histogram) whereas pre‐incubation with buffer containing humAb 1B1 inhibited the hfH binding (green histogram). In panels B and C (testing bacteria expressing variant 1.1 or 3.28), the equal florescence intensity of green and red histograms denotes that pre‐incubation with humAb had no effect on hfH binding.

It is relatively unusual to identify an anti‐fHbp mAb that, in the absence of additional mAbs, is potent in the human complement SBA (hSBA) assay.^23^, ^43^ Indeed, to date, only three highly similar humAbs (4F9, 4B3, and 3G7) have alone shown high potency in the hSBA assay. Previously, we determined the structure of fHbp variant 1 bound to humAb 4B3, revealing that 4B3 bound to a site on fHbp that prevented the simultaneous binding of hfH, thus, at least in part, explaining the high potency of humAb 4B3.^23^ Therefore, we sought to understand whether humAb 1B1 could similarly compete with hfH for binding to fHbp on live meningococci. Using flow cytometry, we found that humAb 1B1 was indeed effective at preventing the binding of hfH to MenB strain M08‐0240104 (expresses variant 2) but had no detectable impact on the binding of hfH to MenB strains MC58 or M1239 that express fHbp variants 1.1 and 3.28, respectively (Figure 3). These findings suggest that humAb 1B1 binds specifically to a region of fHbp variant 2 that overlaps with the binding site of hfH and this competitive binding capability may underlie the high potency of 1B1 in SBA assays.

### 3.4 Wild type fHbp variant 2 structure reveals similarities and differences with known fHbp structures

To deepen our understanding of its hitherto only partly characterized structure, we attempted to crystallize wild type fHbp variant 2. We failed to crystallize the protein alone, likely due to the known low thermostability of its N‐terminal domain (NTD; T_m_ ~ 42°C).^25^, ^27^ Consequently, we sought to promote crystallization by first forming the complex of wild type fHbp variant 2 with Fab 1B1 (the monovalent Fab form of humAb 1B1), the structure of which had not been previously determined.Strongly diffracting crystals of the complex were obtained, and the structure was solved by the MR technique and refined at 2.4 Angstrom (Å) resolution, with the *R*‐factor and free *R*‐factor converging at 20.5% and 26.9%, respectively (Table 2). The asymmetric unit contained three copies of the Fab‐antigen complex (hence, nine polypeptide chains in total) wherein all copies were essentially identical. The final refined models covered the entire Fab heavy and light chain sequences, and the fHbp variant 2 sequence from residue D76‐E261 (except loop residues 121–130, also previously unobserved in fHbp variant 1 structures, likely due to high flexibility of this loop^23^). Intriguingly, no electron density was observed for fHbp variant 2 residues up to N75, suggesting this N‐terminal region was disordered in the crystal. This new data expands our understanding of the wild type fHbp variant 2 structure, while the enigmatic N‐terminal region still remains partially undefined.

**TABLE 2:** Data collection and refinement statistics for the fHbp variant 2/humAb 1B1 co‐crystal structure. fHbp (v2): 1B1 complex (PDB code 8BK2).

Wavelength	0.966
Resolution range	49.66–2.41 (2.49–2.41)
Space group	P1211
Unit cell	77.15135.64121.53 90105.18 90
Total reflections	321,786 (33058)
Unique reflections	92,813 (9128)
Multiplicity	3.5 (3.6)
Completeness (%)	99.7 (99.9)
I/sigma (I)	10.0 (1.1)
CC1/2	0.997 (0.494)
Wilson B‐factor	0.0656 (1.082)
R‐merge	0.066 (1.17)
R‐meas	0.078 (1.38)
Reflections used in refinement	92,645 (9179)
Reflections used for R‐free	4732 (453)
R‐work	0.2050 (0.3568)
R‐free	0.2693 (0.4282)
Number of non‐hydrogen atoms	13,791
Macromolecules	13,495
Ligands	194
Protein residues	1798
RMS (bonds)	0.008
RMS (angles)	0.99
Ramachandran favored (%)	94.78
Ramachandran allowed (%)	4.65
Ramachandran outliers (%)	0.57
Rotamer outliers (%)	2.06
Clashscore	18.74
Average B‐factor	65.97
Macromolecules	66.14
Ligands	64.74
Solvent	55.72

*Note*: Statistics for the highest‐resolution shell are shown in parentheses. Rsym = S hkl S i jIi (hkl) − <I (hkl) >j/S hkl S i Ii (hkl). Rwork = SjjF (obs)j – jF (calc)jj/S jF (obs)j. Rfree is the same as for Rwork but calculated for 5% of the total reflections that were chosen at random and omitted from refinement.

We compared our wild type fHbp variant 2 structure with previously reported fHbp structures. As expected, the structure showed moderate similarity with fHbp variant 3 (overall alpha‐carbon atom root mean square deviation [rmsd] 0.83 Å), high similarity with variant 1 (rmsd 0.67 Å), higher similarity with the unnatural double‐mutant stabilized form of variant 2 (rmsd 0.58 Å),^26^ and very high similarity in the available C‐terminal domain (CTD) sub‐region of wild type variant 2 (rmsd 0.38 Å).^25^ Only upon determination of our wild type fHbp variant 2 structure was it possible to observe differences in the NTD structure derived from previous genetic engineering efforts performed to create the double‐mutated stabilized variant 2 (pdb 4z3t).^26^ Specifically, the wild type NTD β‐strand spanning S133‐G141 makes fewer contacts to the neighboring CTD than the corresponding β‐strand R127‐G136 in the engineered stabilized mutant. This structural difference together with disorder in wild type NTD residues 1–75 may underlie the low thermostability of the wild type variant 2.

### 3.5 Co‐crystal structure reveals fHbp variant 2 epitope targeted by humAb 1B1

**FIGURE 4:**
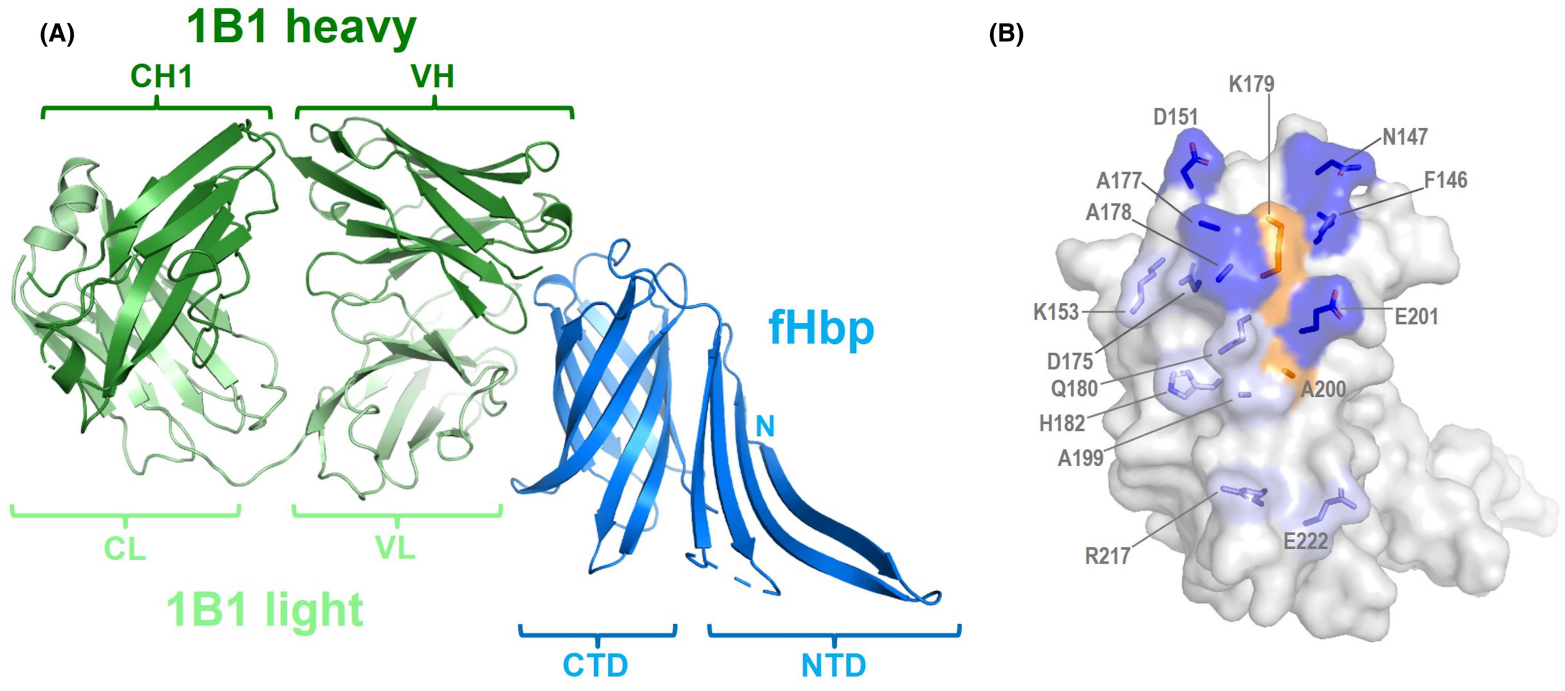
Co‐crystal structure of Fab 1B1 bound to fHbp variant 2 reveals epitope detail. (A) Fab 1B1 heavy and light chains (dark green, light green, respectively) in contact with fHbp (blue). Cartoon labels show variable heavy and light chain regions (VH, VL), constant heavy and light chains (CH1, CL), and fHbp N‐terminal and C‐terminal domains (NTD, CTD). (B) Semi‐transparent surface of fHbp (gray) with the following color scheme: dark blue/light blue patches show fHbp residues (side chain sticks shown) used to bind Fab 1B1 heavy/light chain via H‐bonds and salt bridges; orange/yellow patches show residues making van der Waals' interactions with heavy/light chain. Compared to panel A, the image is enlarged and rotated ~90° around the vertical plane (Fab 1B1 is not shown in panel B).

The co‐crystal structure of wild type fHbp variant 2 bound to Fab 1B1 revealed all the molecular determinants at the epitope/paratope interface. The overall structure of the complex shows Fab 1B1 projecting all six complementarity determining regions (CDRs) onto a convex surface‐exposed region of the fHbp CTD β‐barrel, while the fHbp NTD does not contribute to the interaction (Figure 4A). Thirteen fHbp CTD residues are directly involved in binding to Fab 1B1 via 14 hydrogen bonds and 2 salt‐bridges (Figure 4B), while 6 fHbp residues make van der Waals' interactions with the Fab. Altogether, the Fab covers a surface area on fHbp of 744 Å^2^, a typical size for an epitope.^44^, ^45^

**FIGURE 5:**
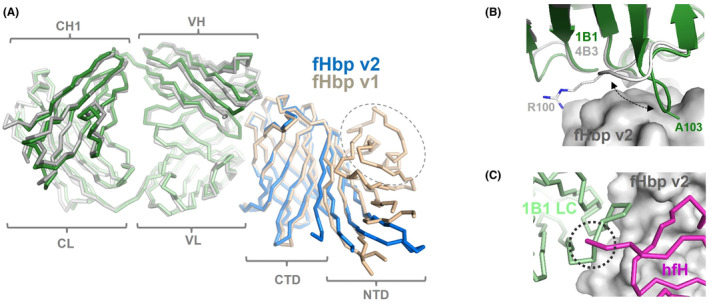
Similarities and differences of Fabs 1B1 (variant 2‐specific) and 4B3 (cross‐reactive) in binding to fHbp. (A) Fab 1B1 (green) and Fab 4B3 (gray) share the same angle of approach to fHbp variant 1 (wheat) and variant 2 (blue). The dashed oval shows the small N‐terminal region of variant 1 which is unobserved in our electron density maps of variant 2, likely due to local disorder. (B) The tertiary structures of Fabs 1B1 and 4B3 are highly superimposable (rmsd 0.54 Å for the VH regions) but the CDR3 loops diverge in opposite directions (see black dashed arrows), enabling the Fabs to engage slightly different epitopes. By sequence alignment, A103 and R100 are corresponding residues, but differ in spatial organization by 4.3 Angstroms, highlighting their different CDRH3 conformations (C) Superposition of the fHbp/1B1 and fHbp/hfH structures (overall rmsd 1.6 Å) shows how the N‐terminal region of hfH (magenta) and the CDR L2 loop of 1B1 (pale green) would clash (dotted circle) upon binding fHbp variant 2 (gray surface), thus preventing simultaneous binding.

It is notable that humAb 1B1 binds to fHbp variant 2 in a region highly similar to where the cross‐protective humAb 4B3 binds to fHbp variant 1, suggesting that this might be a particularly immunogenic region of fHbp (Figure 5A). Of the fifteen total variant 2 epitope residues identified (Figure 4B), 12 corresponding variant 1 residues are present in the 4B3 epitope. However, while the two Fabs approach fHbp with very similar orientations, their CDR H3 loops have very different conformations and engage some different epitope residues (Figure 5B).

The structure also suggested the potential molecular basis for the high functional activity of humAb 1B1. In short, by in silico superposition of the fHbp structures of the variant 2/1B1 complex and the variant 1/hfH complex,^17^ we observed that 1B1 and hfH could not compatibly bind to fHbp at the same time, due to steric hindrance. In particular, the CDR L2 of the 1B1 light chain would clash with the N‐terminal region of hfH domain 6 (Figure 5C).

### 3.6 An epitope hotspot drives the antigen–antibody interaction

**FIGURE 6:**
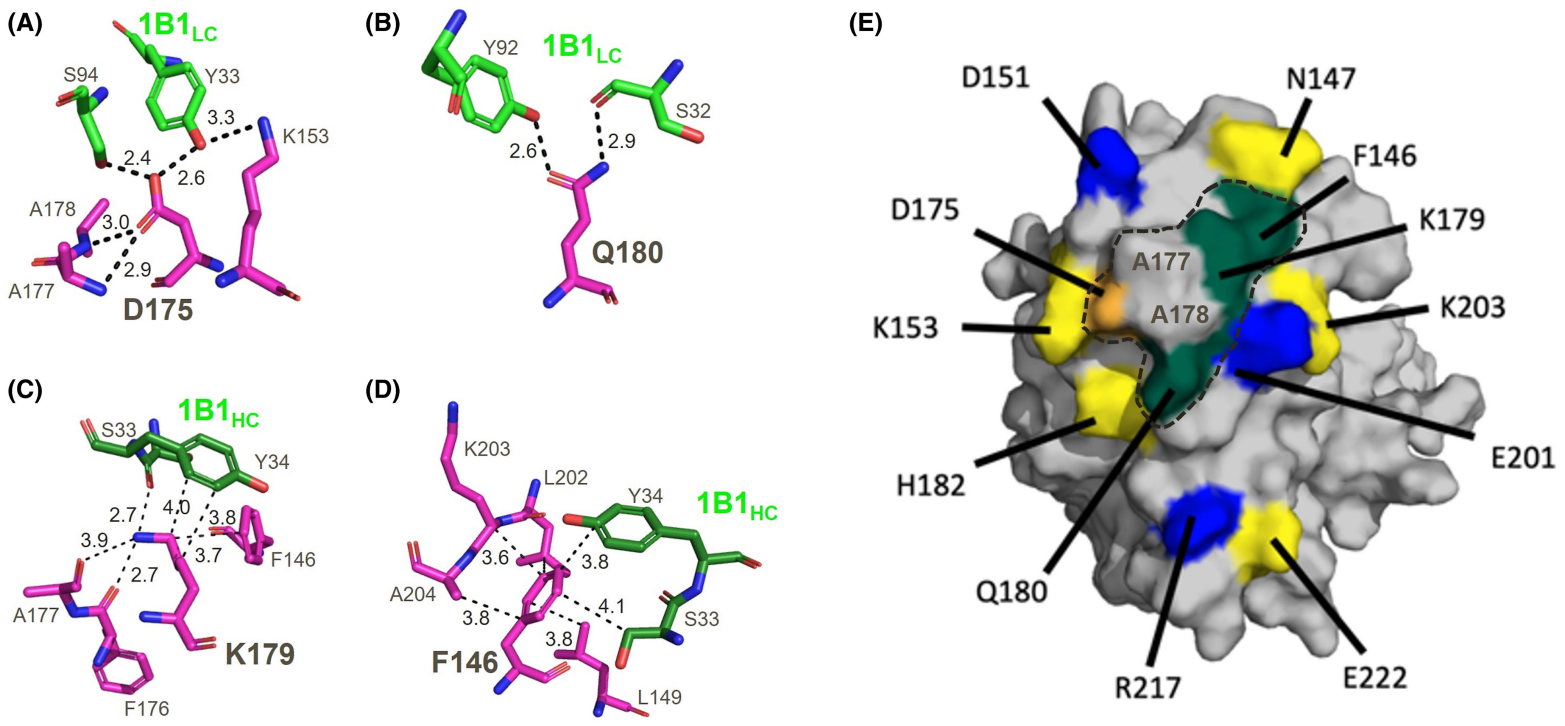
Experimentally verified molecular determinants of the interaction between humAb 1B1 (green) and fHbp variant 2 (magenta). (A) fHbp D175 is central to a multi‐valent H‐bond network, with two H‐bonds to Y33 and S94 on the Fab 1B1 light chain (1B1_LC_); (B) fHbp Q180 makes two H‐bond contacts to S32 and Y92 on 1B1_LC_; (C) fHbp K179 forms one H‐bond with S33 and van der Waals (VdW) interactions with Y34 on 1B1 heavy chain (1B1_HC_); (D) fHbp F146 sits in a hydrophobic pocket on fHbp and makes VdW interactions with S33 and Y34 on 1B1_HC_; (E) Surface plot of fHbp variant 2 (gray) with epitope residues colored according to fold‐increase in K_D_ when mutated to alanine (orange, 200×; teal, 10×; blue, 4×; yellow, <2×). The epitope hotspot lies within the dashed‐line circle; the interface with 1B1 includes A177 and A178 which make VdW interactions that support binding; these residues were obviously not amenable to the alanine scanning approach.

To better understand the antigen–antibody interaction, we performed an extensive alanine scanning study, in which all eleven non‐alanine fHbp epitope residues were individually mutated to alanine. Mutated fHbp proteins were purified and tested via BLI assay for binding to humAb 1B1. The different alanine mutations had varying effects on the strength of the interaction, ranging from essentially conferring no change, up to a 200‐fold decrease in binding affinity (Table 3, left). Four individual mutations resulted in a relatively large loss of affinity (K_D_ values increased ≥10‐fold), indicating important roles in binding for residues F146, K179, Q180, and especially D175. These key mutations likely abolished one or more H‐bonds and/or reduced van der Waals interactions with the 1B1 light chain CDRs L1 and L3 and the heavy chain CDR H1 (Figure 6). These mutational data suggest a hotspot in the “centre/upper‐right region” of the epitope (Figure 6E) as the key antigenic area driving the strength of the antigen–antibody interaction. Intriguingly, five of these eleven mutations also decreased the binding affinity of fHbp variant 2 to humAb 4B3 (Table 3, right), though with a different rank order of impact on binding strength. Notably, despite its proximity to the hotspot residues, the D175A mutation surprisingly had no impact on binding to humAb 4B3. These data demonstrate that while the two humAbs 1B1 and 4B3 appear to bind the same site on fHbp, they achieve high binding affinity in different ways.

**TABLE 3:** Impact of alanine point mutations in fHbp variant 2 on its binding to humAbs 1B1 (left) or 4B3 (right).

fHbp var 2 mutant	1B1 affinity		4B3 affinity	
	K_D_ (nM)	Fold change	K_D_ (nM)	Fold change
*Wild type*	0.24 ± 0.03	n/a	0.002	n/a
N147A	0.26 ± 0.03	1.1	0.001	0.5
H182A	0.38 ± 0.11	1.6	0.002	1.0
K153A	0.45 ± 0.10	1.9	0.023 ± 0.04	11.5
E222A	0.50 ± 0.11	2.1	0.001	0.5
R217A	0.96 ± 0.15	4.0	0.001	0.5
D151A	1.13 ± 0.20	5	0.001	0.5
E201A	1.71 ± 0.44	7	0.042 ± 0.04	21
F146A	2.36 ± 0.25	10	0.739 ± 0.06	370
K179A	3.39 ± 0.47	14	4.8 ± 0.11	2390
Q180A	4.13 ± 0.75	17	1.35 ± 0.05	677
D175A	49.09 ± 32.35	205	0.002	1.0

*Note*: Data are tabulated and ranked by the “fold change” in K_D_ for binding to 1B1 when compared to the wild type fHbp variant 2.16. BLI measurements were performed at least in duplicate and the mean and standard deviation (s.d.) are reported. An accurate s.d. could not be reliably determined for samples binding to 4B3 with very high affinity (K_D_ 1–2 pM) due to the difficulty of BLI measurements for extremely tight interactions where dissociation occurs only very slowly.

### 3.7 Molecular basis for fHbp variant 2 specificity of humAb 1B1

Interestingly, all four key epitope hotspot residues (F146, D175, K179, and Q180), are identically conserved in fHbp variants 1.1 and 3.28, suggesting that, while important for the strength of binding, they are unlikely to account for the specificity of humAb 1B1 for fHbp variant 2. Rather, the epitope peripheral residue D151, for which the alanine mutation caused approximately five‐fold lower binding affinity to humAb 1B1, is a notable epitope residue not at all conserved in both variant 1 and 3 (it is replaced by glycine, which has no side chain). Also, H182 is mutated to N and Y in variants 1 and 3, respectively, but since the H182A mutation had little effect on binding strength, this residue seemed less likely to be important for specificity. Overall, these observations suggested that the acidic side chain of D151 may provide the crucial interactions specific for binding to humAb 1B1 (namely, two H‐bonds to Ser56 and Ser58 in CDR H2). Further, given the shape complementarity of the epitope/paratope interface in the hotspot region involving Ala177 and Ala178 (Figure 6E), it was conceivable that their switch to the bulkier Thr and Lys residues in variant 3 might obstruct binding. To explore these hypotheses, we first made a series of mutations in fHbp variant 3, attempting to create gain‐of‐function mutants that could bind to humAb 1B1. Using the BLI assay to test humAb 1B1 interactions with fHbp variant 3 mutants, we found that single mutants G151D and T177A were still unable to bind 1B1, whereas the double mutant T177A/K178A and the triple mutant G151D/T177A/K178A exhibited quite tight binding (Table 4, upper), albeit ~50‐fold weaker than variant 2. These data suggest the main reason wild type fHbp variant 3 does not bind to humAb 1B1 is due to steric hindrance at position K178.

**TABLE 4:** Impact of alanine point mutations in fHbp variants 1 and 3 on binding to humAb 1B1.

fHbp mutant	K_D_ (nM)
*Var3* G151D	No binding
*Var3* T177A	No binding
*Var3* T177A, K178A	14.8 ± 3.1
*Var3* G151D, T177A, K178A	12.1 ± 1.5
*Var1* E151D, delG152	269 ± 150
*Var1* E151D, delG152, D201E	32.2 ± 3.8
*Var1* E151D, delG152, D201E, L217R	14.2 ± 1.4

Since fHbp variant 1 contains Alanines like A177/A178 in variant 2, a different reason must underlie its inability to binding humAb 1B1. Indeed, compared to variant 2, while variant 3 replaces D151 simply with glycine, in variant 1 there is a Glu‐Gly dipeptide motif, in a conformation likely incompatible with forming bonds to 1B1. To explore these observations experimentally, we also tested fHbp variant 1 mutants, and found that moderate binding to humAb 1B1 could be achieved by the simultaneous E151D mutation and G152 deletion (Table 4, lower). Binding affinity was further increased by D201E and L217R mutations, which flank the epitope hotspot (Figure 4B). Collectively, these mutational studies of fHbp variants 1, 2 and 3 help to explain the variant 2 specificity of humAb 1B1.

### 3.8 humAbs 1B1 and 4B3 have different germline origins yet bind equivalent epitopes

The high degree of structural similarity exhibited by the 1B1/variant 2 and 4B3/variant 1 complexes (Figure 5A) prompted us to ask whether the two humAbs share similar sequences despite deriving from different donors. We analyzed the nucleotidic sequences of both humAbs using NCBI *igblastn* version 1.18.0.^46^ The VDJ gene sequences revealed that the heavy chains of humAbs 1B1 and 4B3 are both IgG1 isotype but have different genetic origins, while the light chains (IgK type) are more similar (Table 5). The 1B1 and 4B3 heavy chain variable regions (VH) share only 74% amino acid sequence identity, while their light chain variable regions (VL) share 83% sequence identity. Interestingly, while all six CDRs contact the antigen in both complexes, it is the heavy chain of 1B1 that makes the majority of contacts with the epitope hotspot on fHbp variant 2 (Figure 6E), thus further explaining the different binding capabilities of the two humAbs. Nucleotidic sequence alignments suggested that overall humAbs 1B1 and 4B3 heavy chains shared 99% and 88% sequence identity with their inferred V‐gene germline precursors (Table 5), highlighting diverse degrees of somatic hypermutation of these two humAbs, enabling their distinct specificities despite their remarkably similar structures, epitope regions, and angles of approach to the antigens.

**TABLE 5:** IgG variable region sequence analyses of humAbs 1B1 and 4B3.

humAb name	Heavy chain					Light chain			
	V	D	J	IgCH	% identity VH V‐gene GL	V	J	IgCL	% identity VL V‐gene GL
1B1	IGHV3‐48*04	IGHD6‐13*01	IGHJ4*02	IgG1	98.98	IGKV3‐20*01	IGKJ1*01	IgK	96.90
4B3	IGHV3‐11*01	IGHD3‐16*01	IGHJ4*02	IgG1	88.10	IGKV3‐20*01	IGKJ2*01	IgK	90.24

*Note*: The colors shades (dark and light gray) need only to better distinguish Heavy chain respect to Light chain.

## 4 DISCUSSION

*N*. *meningitidis* is a globally important pathogen causing invasive meningococcal disease, often with severe sequelae and fatal in 10%–20% of cases. Two highly effective serogroup B meningococcal vaccines are now available, containing fHbp variants 1 and 3 (MenB‐fHbp vaccine), or fHbp variant 1 and other antigens (4CMenB vaccine). Since the breadth of protection afforded by such vaccines might be further increased by the inclusion of additional, diverse fHbp antigens, we sought to characterize the immunologically distinct fHbp variant 2. The latter is relatively uncharacterized and our vision was that determination of its structure and antigenicity might enable the design and development of improved vaccines.

Since we, like others previously, were not able to crystallize the wild type fHbp variant 2 protein alone, we sought a high affinity variant 2‐specific mAb, the Fab form of which might “chaperone” crystallization. Such co‐crystallization methods have been successfully used previously.^47^ In an earlier study of numerous humAbs, we had preliminarily identified an apparent variant 2‐specific Fab, termed 1B1.^24^ In the present work, we performed a detailed study of 1B1, both in IgG1 and Fab formats. In doing so we have characterized, to the best of our knowledge, the first fHbp variant 2‐specific humAb. We have revealed at the molecular level the ability of 1B1 to compete the interaction of fHbp with human fH, thus increasing the susceptibility of the bacteria to complement‐mediated bacteriolysis and explaining its mechanism of action as a highly potent bactericidal antibody. Moreover, Fab 1B1 was indeed able to chaperone the crystallization of wild type fHbp variant 2, enabling structure determination at high resolution.

Analysis of the paratope/epitope interface formed between Fab 1B1 and fHbp variant 2 revealed the basis of its antigen specificity. A key molecular determinant of the interaction is an H‐bond between 1B1‐Ser56 and fHbp variant 2 Asp151, the latter being part of the 150‐PDGKA‐154 loop. A crucial local difference is the insertion of a second Gly in fHbp variant 1 (PEG**G**RA) which distorts the loop structure and likely prevents the variant 1 Glu residue from making the productive interaction with 1B1‐Ser56. This and other observations described above explain the antigen specificity of humAb 1B1 and were sufficient to inform the design of mutations in fHbp variants 1 and 3 that conferred gain‐of‐function to enable binding to humAb 1B1.

Unexpectedly, Fab 1B1 was found to bind fHbp in a manner extremely similar to that of the cross‐reactive Fab 4B3. Notwithstanding an almost identical angle of approach used by the two Fabs, and high structural similarity of their framework regions (Figure 5A), Fab 1B1 did not bind to wild type fHbp variants 1 or 3, while Fab 4B3 is cross‐reactive. Analyses of the sequences of these two humAbs suggests they have different origins, and yet converged upon a similar spatial solution to engaging this key antigen. Indeed, not only do they bind to similar epitopes, both Fabs can also prevent the binding of human fH to the antigen. This finding is particularly relevant, since the 1B1 structure hereby provides a second example (after 4B3) of a humAb able to bind the hfH binding site, confirming that this surface region on fHbp is at least partially or temporarily exposed and immunogenic in humans. The latter topic was previously debated, since it was thought that the region was masked from the immune system due to binding of human fH. The data we present here support the hypothesis that the site is accessible and immunogenic, perhaps because the circulating human fH concentration is simply insufficient to occupy all possible sites on fHbp and completely shield them from the immune system.

Despite having provided the first structural and functional insights into a human mAb with specificity for fHbp variant 2, there remain some unanswered questions. For example, despite Fab 1B1 having aided crystallization of fHbp variant 2, there was a notable absence of some N‐terminal fHbp residues (V9‐N75) in the crystallographic electron density maps. This prompts the question as to whether the N‐terminal region is naturally disordered or, rather, was destabilized by the presence of the mAb and/or the buffer conditions used for crystallization, or a combination of both factors. The former explanation seems more likely since several studies of fHbp variant 2 in solution have indicated a low thermal stability of its N‐terminal domain. It is unclear from an evolutionary viewpoint why the variant 2 protein should have maintained this low stability, which plausibly leads to its more rapid proteolytic turnover on the bacterial surface and consequent loss of function in situ. Further, our studies were limited to typical exemplars of the fHbp variants 1, 2 and 3 (namely, variants 1.1, 2.16, and 3.28), for which humAb 1B1 displayed distinct specificity for variant 2. Considering that there are over 1380 distinct fHbp amino sequences, it is possible that some sub‐variants of variants 1 or 3 may display modest affinity for humAb 1B1. Finally, it was unexpected to obtain a mAb with specificity to fHbp variant 2. We speculate that the individual donor from whom we identified mAb 1B1 might have been previously infected with a meningococcal strain expressing fHbp variant 2 and/or our in vitro assays had features that enabled selection of such a B cell. Further studies will be required to address such questions.

In summary, our structural and functional investigations have shed additional light on the nature of the human immune response to meningococcal fHbp antigens. Specifically, the generation of new data on the relatively uncharacterized variant 2 fHbp protein should enable the design of alternative novel candidate vaccine antigens, potentially by rational design to improve molecular stability while ensuring the presence of demonstrated protective epitopes capable of eliciting potent bactericidal antibodies. Moreover, variant 2‐specific antibodies may be useful for the characterization of this specific antigen in the context of multi‐antigen vaccines. Overall, the data presented may be of particular benefit to support the development of meningococcal vaccines, and more broadly demonstrate the value of combining human immunology and structural biology studies to deepen our understanding of pathology and vaccinology.

## AUTHOR CONTRIBUTIONS

D.V., C.C.C., R.C., F.B., L.C., J.L., Y.H., A.B.C., M.J.B. designed and/or conducted the studies, including data collection and data analysis. D.V., C.C.C., L.S., and M.J.B. prepared the manuscript. All authors reviewed and approved the final manuscript. All authors had full access to the study data.

## FUNDING INFORMATION

This study was performed at GSK Vaccines Srl (Siena, Italy) and at GSK Vaccines (Rockville, US), both locations are part of the GSK group of companies. FB was the recipient of a PhD fellowship at the University of Florence.

## CONFLICT OF INTEREST STATEMENT

All authors were employees of the GSK group of companies at the time of the study, with the exception of FB, who held a PhD studenship at the University of Florence, Italy. The authors DV and MJB are named inventors on patent applications related to fHbp polypeptides. MJB, DM report ownership of GSK shares.

## Data Availability

The data that support the findings of this study are available in the methods and/or supplementary material of this article.

## References

[1] ECDC . European Centre for Disease Prevention and Control. Invasive meningococcal disease. ECDC Ann Epidemiol Rep. 2017;27:1‐10.

[2] Rosenstein NE , Perkins BA , Stephens DS , Popovic T , Hughes JM . Meningococcal disease. N Engl J Med. 2001;344:1378‐1388. 11333996 10.1056/NEJM200105033441807

[3] Pace D , Pollard AJ . Meningococcal disease: clinical presentation and sequelae. Vaccine. 2012;30(Suppl. 2):B3‐B9. 22607896 10.1016/j.vaccine.2011.12.062

[4] Olbrich KJ , Muller D , Schumacher S , Beck E , Meszaros K , Koerber F . Systematic review of invasive meningococcal disease: sequelae and quality of life impact on patients and their caregivers. Infect Dis Ther. 2018;7:421‐438. 30267220 10.1007/s40121-018-0213-2 PMC6249177

[5] Pace D , Pollard AJ , Messonier NE . Quadrivalent meningococcal conjugate vaccines. Vaccine. 2009;27(Suppl. 2):B30‐B41. 19477560 10.1016/j.vaccine.2009.05.003

[6] Ruiz Garcia Y , Abitbol V , Pellegrini M , Bekkat‐Berkani R , Soumahoro L . A decade of fighting invasive meningococcal disease: a narrative review of clinical and real‐world experience with the MenACWY‐CRM conjugate vaccine. Infect Dis Ther. 2021;11:639‐655. 10.1007/s40121-021-00519-2 PMC8481757 34591258

[7] Seib KL , Scarselli M , Comanducci M , Toneatto D , Masignani V . *Neisseria meningitidis* factor H‐binding protein fHbp: a key virulence factor and vaccine antigen. Expert Rev Vaccines. 2015;14:841‐859. 25704037 10.1586/14760584.2015.1016915

[8] Serruto D , Bottomley MJ , Ram S , Giuliani MM , Rappuoli R . The new multicomponent vaccine against meningococcal serogroup B, 4CMenB: immunological, functional and structural characterization of the antigens. Vaccine. 2012;30(Suppl. 2):B87‐B97. 22607904 10.1016/j.vaccine.2012.01.033 PMC3360877

[9] Zlotnick GW , Jones TR , Liberator P , et al. The discovery and development of a novel vaccine to protect against *Neisseria meningitidis* serogroup B disease. Hum Vaccin Immunother. 2015;11:5‐13. 25483509 10.4161/hv.34293 PMC4514153

[10] Isitt C , Cosgrove CA , Ramsay ME , Ladhani SN . Success of 4CMenB in preventing meningococcal disease: evidence from real‐world experience. Arch Dis Child. 2020;105:784‐790. 32029437 10.1136/archdischild-2019-318047

[11] Masignani V , Pizza M , Moxon ER . The development of a vaccine against meningococcus B using reverse vaccinology. Front Immunol. 2019;10:751. 31040844 10.3389/fimmu.2019.00751 PMC6477034

[12] Watson PS , Novy PL , Friedland LR . Potential benefits of using a multicomponent vaccine for prevention of serogroup B meningococcal disease. Int J Infect Dis. 2019;85:22‐27. 31102824 10.1016/j.ijid.2019.05.019

[13] Principato S , Pizza M , Rappuoli R . Meningococcal factor H‐binding protein as immune evasion factor and vaccine antigen. FEBS Lett. 2020;594:2657‐2669. 32298465 10.1002/1873-3468.13793

[14] Cantini F , Veggi D , Dragonetti S , et al. Solution structure of the factor H‐binding protein, a survival factor and protective antigen of *Neisseria meningitidis* . J Biol Chem. 2009;284:9022‐9026. 19196709 10.1074/jbc.C800214200 PMC2666550

[15] Cendron L , Veggi D , Girardi E , Zanotti G . Structure of the uncomplexed *Neisseria meningitidis* factor H‐binding protein fHbp (rLP2086). Acta Crystallogr Sect F Struct Biol Cryst Commun. 2011;67:531‐535. 10.1107/S1744309111006154 PMC3087634 21543855

[16] Mascioni A , Bentley BE , Camarda R , et al. Structural basis for the immunogenic properties of the meningococcal vaccine candidate LP2086. J Biol Chem. 2009;284:8738‐8746. 19103601 10.1074/jbc.M808831200 PMC2659232

[17] Schneider MC , Prosser BE , Caesar JJ , et al. *Neisseria meningitidis* recruits factor H using protein mimicry of host carbohydrates. Nature. 2009;458:890‐893. 19225461 10.1038/nature07769 PMC2670278

[18] Madico G , Welsch JA , Lewis LA , et al. The meningococcal vaccine candidate GNA1870 binds the complement regulatory protein factor H and enhances serum resistance. J Immunol. 2006;177:501‐510. 16785547 10.4049/jimmunol.177.1.501 PMC2248442

[19] Schneider MC , Exley RM , Chan H , et al. Functional significance of factor H binding to *Neisseria meningitidis* . J Immunol. 2006;176:7566‐7575. 16751403 10.4049/jimmunol.176.12.7566

[20] Jolley KA , Bray JE , Maiden MCJ . Open‐access bacterial population genomics: BIGSdb software, the PubMLST.org website and their applications. Wellcome Open Res. 2018;3:124. 30345391 10.12688/wellcomeopenres.14826.1 PMC6192448

[21] Masignani V , Comanducci M , Giuliani MM , et al. Vaccination against *Neisseria meningitidis* using three variants of the lipoprotein GNA1870. J Exp Med. 2003;197:789‐799. 12642606 10.1084/jem.20021911 PMC2193853

[22] Lopez‐Sagaseta J , Beernink PT , Bianchi F , et al. Crystal structure reveals vaccine elicited bactericidal human antibody targeting a conserved epitope on meningococcal fHbp. Nat Commun. 2018;9:528. 29410413 10.1038/s41467-018-02827-7 PMC5802752

[23] Veggi D , Bianchi F , Santini L , et al. 4CMenB vaccine induces elite cross‐protective human antibodies that compete with human factor H for binding to meningococcal fHbp. PLoS Pathog. 2020;16:e1008882. 33007046 10.1371/journal.ppat.1008882 PMC7556464

[24] Bianchi F , Veggi D , Santini L , et al. Cocrystal structure of meningococcal factor H binding protein variant 3 reveals a new crossprotective epitope recognized by human mAb 1E6. FASEB J. 2019;33:fj201900374R‐fj201912111R. 10.1096/fj.201900374R PMC6902690 31442074

[25] Johnson S , Tan L , van der Veen S , et al. Design and evaluation of meningococcal vaccines through structure‐based modification of host and pathogen molecules. PLoS Pathog. 2012;8:e1002981. 23133374 10.1371/journal.ppat.1002981 PMC3486911

[26] Konar M , Pajon R , Beernink PT . A meningococcal vaccine antigen engineered to increase thermal stability and stabilize protective epitopes. Proc Natl Acad Sci USA. 2015;112:14823‐14828. 26627237 10.1073/pnas.1507829112 PMC4672778

[27] Malito E , Faleri A , Lo Surdo P , et al. Defining a protective epitope on factor H binding protein, a key meningococcal virulence factor and vaccine antigen. Proc Natl Acad Sci USA. 2013;110:3304‐3309. 23396847 10.1073/pnas.1222845110 PMC3587270

[28] Graham BS , Gilman MSA , McLellan JS . Structure‐based vaccine antigen design. Annu Rev Med. 2019;70:91‐104. 30691364 10.1146/annurev-med-121217-094234 PMC6936610

[29] Malito E , Carfi A , Bottomley MJ . Protein crystallography in vaccine research and development. Int J Mol Sci. 2015;16:13106‐13140. 26068237 10.3390/ijms160613106 PMC4490488

[30] Andreano E , Seubert A , Rappuoli R . Human monoclonal antibodies for discovery, therapy, and vaccine acceleration. Curr Opin Immunol. 2019;59:130‐134. 31450054 10.1016/j.coi.2019.07.005

[31] Beernink PT , Granoff DM . Bactericidal antibody responses induced by meningococcal recombinant chimeric factor H‐binding protein vaccines. Infect Immun. 2008;76:2568‐2575. 18362128 10.1128/IAI.00033-08 PMC2423104

[32] Vu DM , Pajon R , Reason DC , Granoff DM . A broadly cross‐reactive monoclonal antibody against an epitope on the n‐terminus of meningococcal fHbp. Sci Rep. 2012;2:341. 22461972 10.1038/srep00341 PMC3314305

[33] Kabsch W . Xds. Acta Crystallogr D Biol Crystallogr. 2010;66(Pt 2):125‐132. doi:10.1107/S0907444909047337 20124692 PMC2815665

[34] Evans PR , Murshudov GN . How good are my data and what is the resolution? Acta Crystallogr D Biol Crystallogr. 2013;69(Pt 7):1204‐1214. doi:10.1107/S0907444913000061 23793146 PMC3689523

[35] Winn MD , Ballard CC , Cowtan KD , et al. Overview of the CCP4 suite and current developments. Acta Crystallogr D Biol Crystallogr. 2011;67(Pt 4):235‐242. doi:10.1107/S0907444910045749 21460441 PMC3069738

[36] McCoy AJ , Grosse‐Kunstleve RW , Adams PD , Winn MD , Storoni LC , Read RJ . Phaser crystallographic software. J Appl Crystallogr. 2007;40(Pt 4):658‐674. doi:10.1107/S0021889807021206 19461840 PMC2483472

[37] Emsley P , Cowtan K . Coot: model‐building tools for molecular graphics. Acta Crystallogr D Biol Crystallogr. 2004;60(Pt 12 Pt 1):2126‐2132. doi:10.1107/S0907444904019158 15572765

[38] Afonine PV , Grosse‐Kunstleve RW , Echols N , et al. Towards automated crystallographic structure refinement with phenix.refine. Acta Crystallogr D Biol Crystallogr. 2012;68(Pt 4):352‐367. doi:10.1107/S0907444912001308 22505256 PMC3322595

[39] Berman H , Henrick K , Nakamura H . Announcing the worldwide Protein Data Bank. Nat Struct Biol. 2003;10(12):980. doi:10.1038/nsb1203-980 14634627

[40] Frasch CE , Borrow R , Donnelly J . Bactericidal antibody is the immunologic surrogate of protection against meningococcal disease. Vaccine. 2009;27(Suppl. 2):B112‐B116. 19464093 10.1016/j.vaccine.2009.04.065

[41] Poolman JT , Richmond P . Multivalent meningococcal serogroup B vaccines: challenges in predicting protection and measuring effectiveness. Expert Rev Vaccines. 2015;14:1277‐1287. 26204792 10.1586/14760584.2015.1071670

[42] Brunelli B , Del Tordello E , Palumbo E , et al. Influence of sequence variability on bactericidal activity sera induced by Factor H binding protein variant 1.1. Vaccine. 2011;29(5):1072‐1081. doi:10.1016/j.vaccine.2010.11.064 21130753

[43] Giuliani M , Bartolini E , Galli B , et al. Human protective response induced by meningococcus B vaccine is mediated by the synergy of multiple bactericidal epitopes. Sci Rep. 2018;8:3700. 29487324 10.1038/s41598-018-22057-7 PMC5829249

[44] MacRaild CA , Richards JS , Anders RF , Norton RS . Antibody recognition of disordered antigens. Structure. 2016;24:148‐157. 26712277 10.1016/j.str.2015.10.028

[45] Rubinstein ND , Mayrose I , Halperin D , Yekutieli D , Gershoni JM , Pupko T . Computational characterization of B‐cell epitopes. Mol Immunol. 2008;45:3477‐3489. 18023478 10.1016/j.molimm.2007.10.016

[46] Ye J , Ma N , Madden TL , Ostell JM . IgBLAST: an immunoglobulin variable domain sequence analysis tool. Nucleic Acids Res. 2013;41:W34‐W40. 23671333 10.1093/nar/gkt382 PMC3692102

[47] Griffin L , Lawson A . Antibody fragments as tools in crystallography. Clin Exp Immunol. 2011;165:285‐291. 21649648 10.1111/j.1365-2249.2011.04427.x PMC3170977

